# Perceived neighborhood safety related to physical activity but not recreational screen-based sedentary behavior in adolescents

**DOI:** 10.1186/s12889-017-4756-z

**Published:** 2017-09-18

**Authors:** Clare M. Lenhart, Andrew Wiemken, Alexandra Hanlon, Mackenzie Perkett, Freda Patterson

**Affiliations:** 1 East Stroudsburg University, East Stroudsburg, PA USA; 2 University of Pennsylvania, Philadelphia, PA USA; 3 Department of Behavioral Health and Nutrition, College of Health Sciences, University of Delaware, 019 Carpenter Sports Building, Newark, DE USA

## Abstract

**Background:**

A growing proportion of adolescents have poor cardiovascular health behaviors, including low levels of physical activity and high levels of sedentary behavior, thus increasing the likelihood of poor heart health in later years. This study tested the hypothesis that low perceived neighborhood safety would be associated with low levels of physical activity and high levels of recreational sedentary behavior in high-school students.

**Methods:**

Using cross-sectional, weighted data from the 2015 Pennsylvania (USA) State and Philadelphia city Youth Risk Behavior Survey, multivariable logistic regression modeling was used to examine the association between perceived neighborhood safety, and physical activity levels and recreational screen-based sedentary behavior time respectively, while controlling for potential confounders.

**Results:**

After adjustment for other significant correlates of physical activity, students with low perceived neighborhood safety had a 21% reduced odds of being physically active on 5 or more days of the last week as compared to those who felt safe (*p* = 0.044). Perceived safety was not related to sedentary behavior; but sports team participation emerged as a strong correlate of low screen-based sedentary behavior (OR = 0.73, *p* = .002).

**Conclusion:**

These data add to a growing body of work demonstrating the importance of perceived safety with physical activity levels in youth. Sports team participation may be a viable target to reduce screen-based sedentary time.

## Background

Cardiovascular disease is the leading cause of death among adults globally, and in the United States [1, 2]. Stemming adult cardiovascular disease through the promotion of heart health behaviors, such as increased physical activity, and decreased recreational sedentary behavior in youth, is a viable public health approach [3]. Underpinning the importance of this work are data showing that cardiovascular disease and the associated risk factors (e.g., obesity) is on the rise among youth [4–7]. For example, between 2001 and 2009 a 30.5% increase in type 2 diabetes in youth was reported [5]. Approximately three in five 2–19 year olds are overweight or obese [8], while one in five youth aged 8 to 17 years have an adverse lipid concentration and slightly more than 1 in 10 have either borderline high, or high, blood pressure [9]. Combined, less than 1% of adolescents achieve ideal heart health as defined by the American Heart Association, with fewer Black/African American youth meeting the recommendations for physical activity, blood pressure and cholesterol than Non-Hispanic Whites [10, 11].

Ecological models of health behavior have helped elucidate the role of built environment and neighborhood factors on health [12]. In the context of physical activity and sedentary behavior, access and proximity to green space and recreation facilities, safe neighborhoods with low crime as well as streetscape features such as walkable sidewalks have all been significantly associated with increased physical activity and lower sedentary behavior (e.g., screen-based sedentary behavior) in both adults and youth [13–15]. Lower-income, urban neighborhoods tend to be characterized by features not conducive to higher physical activity and lower recreational sedentary behavior such as limited green space, unappealing aesthetics, damaged sidewalks and poor quality recreation facilities [16–18]. Residents of these neighborhoods, regardless of race, have a lower likelihood of engaging in habitual heart health behaviors such as physical activity. For example, Wilson and colleagues reported that in low-income, urban environments, racial differences in physical inactivity were ameliorated with Whites and Blacks having similar odds of low activity levels [19]. Racially diverse, urban populations represent a particularly high-risk group for cardiovascular disease and poor heart health behaviors.

Neighborhood safety, and in particular, perceived neighborhood safety, are distinct environmental constructs that are relevant to urban residents. In adult populations, neighborhood safety has been associated with physical activity in some [20] but not all studies [21]. Perceptions of neighborhood safety may account for this difference [22, 23]. For example, perception of neighborhood safety is a determinant of physical activity, even after adjustment for objective markers of safety such as neighborhood crime [22–24]. Higher levels of perceived safety and access to recreation facilities predicted higher physical activity levels in adults of low but not high socioeconomic status [19]. Given that perceived safety is a salient and highly associated construct of physical activity and to a lesser extent, sedentary behavior, in adults, the same may be true for youth.

To add to this body of research, we used a population sample of adolescents aged 14–18 years (*n* = 4020) to examine the extent to which lower levels of perceived safety was associated with lower levels of physical activity and higher recreational screen-based sedentary behavior. We adjusted for other known determinants of physical activity and sedentary behavior including gender, race/ethnicity [25, 26], poor mental health [27], age [28], overweight [29], sleep [30], fruit and vegetable consumption [31], sports team and physical education class participation [32].

## Methods

### Study sample

Data from the 2015 Centers for Disease Control (CDC) Youth Risk Behaviors Survey (YRBS) administered in the state of Pennsylvania and the School District of Philadelphia were used for this analysis. A two-stage, cluster sample design was used to produce a representative sample of high school students across Pennsylvania. In the first stage, proportional sampling of schools was based on school enrollment size; in the second stage, classroom-level sampling was conducted in which specific classrooms were randomly sampled from a comprehensive list of all classes available in a given required period; all students in sampled classes were eligible to participate [33]. Data for the current analysis include classes from 64 high schools across the state of Pennsylvania and 25 additional high schools from the urban center of Philadelphia. Student response rate from the Pennsylvania state high schools was 64% (*n* = 2899) and from the Philadelphia schools it was 68% (*n* = 1896). Parental consent and student assent were obtained prior to surveying and all study procedures were approved by the Institutional Review Board and by each participating school district (IRB# 21251).

For the present study, students providing complete data for study demographics (age, sex, race), the independent variable of perceived safety, and dependent variables of physical activity and sedentary behavior were retained. The resulting sample, *n* = 4020 (*N* = 474,768), represents 83.8% of all respondents in the original Philadelphia and Pennsylvania samples.

### Instrument

The YRBS is a school-based biennial, self-administered paper-pencil survey of US high school students, administered at the national level by CDC and by states, territories, tribal areas or large urban school districts. Conducted since 1991, the surveys include representative samples of students in grades 9-12 and monitor six categories of priority health-risk behaviors: behaviors relating to injuries and violence, sexual risk behaviors, tobacco use, alcohol and other drug use, unhealthy diet, and physical inactivity [33, 34]. Reliability assessments of the YRBS instrument have demonstrated moderate reliability (mean kappa 61%) [35].

### Measures

The study outcome of physical activity was measured using the single item “During the past 7 days, on how many days were you physically active for a total of at least 60 minutes per day? (Add up all the time you spent in any kind of physical activity that increased your heart rate and made you breathe hard some of the time?)” Responses were dichotomized: participants reporting 5 or more days of moderate physical activity per week were considered physically active [36].

The study outcome of recreational screen-based sedentary behavior was estimated using two questions that queried students to self-report television watching and recreational computer use. The survey items were: “On an average school day, how many hours do you watch TV?” and “On an average school day, how many hours do you play video or computer games or use a computer for something that is not school work?” Seven response options were presented for each question ranging from, “I do not [watch TV/play video or computer games] on an average school day” to “5 or more hours per day.” Responses to both questions were added together and based on a median split of the resulting combined data, those students reporting four or less hours of combined TV and video/computer game usage were compared to those reporting more than 4 h in recreational screen-based sedentary behavior. The main exposure variable of neighborhood safety was assessed using the item, "How often do you feel safe and secure in your neighborhood?" Those responding “always” or “most of the time” were compared to those responding “sometimes,” “rarely,” or “never” [37].

Several health behaviors were assessed using single survey items that were coded dichotomously (yes/no). These behaviors included: current smoking (one or more cigarettes in the last 30 days); consumption of three or more servings of vegetables per day, consumption of two or more servings of fruit per day; adequate sleep (≥ 8 h) on a school night; any sports team participation in the last year; and physical education class participation on at least 1 day per week [33].

Participants’ socio-demographic characteristics including age, sex and ethnicity/race were self-reported. In the current analyses the ethnicity/race items were collapsed to generate the following categories: White, Black or African American, Asian, Hispanic and Other. The “Other” category was comprised of students who self-identified as more than one race, American Indian or Alaskan Native, Native Hawaiian or Other Pacific Islander.

### Statistical analysis

Descriptive statistics were generated for all study variables. Tests of association between each independent variable with the dichotomous outcomes of physical activity and recreational screen-based sedentary behavior were examined using chi-square test of independence. The independent variable of perceived neighborhood safety along with those variables modestly associated with the outcome measures (*p* < 0.2) were entered into separate multivariable logistic regression models to identify variables uniquely associated with physical activity and screen-based sedentary behavior after controlling for other related variables. The multivariable model of physical activity included perceived neighborhood safety, gender, race/ethnicity, academic achievement, weight status, area of residence, sports team participation, sleep, fruit consumption, vegetable consumption, and screen-based sedentary behavior. The multivariable model of screen-based sedentary behavior included perceived neighborhood safety, gender, race/ethnicity, academic achievement, weight status, physical education participation, sports team participation, vegetable consumption, and physical activity. Analyses were run using SPSS version 23. Having achieved necessary overall response rate within Philadelphia and Pennsylvania, defined as ≥60% after multiplying the school response rate and the student response rate, data were weighted to account for student non-response and demographic distributions in each district such that the weight reflects the number of students the respondent represents [33, 34]. Sampling errors were estimated from the primary sampling units and strata and using Taylor series linearization for SPSS version 23 (IBM, New York).

## Results

### Participant characteristics

Approximately half the sample (50.5%) was female, 59.5% were 16 years or older, 70.9% were non-Hispanic white, 15.2% were non-Hispanic black, 8.4% self-identified as Hispanic and 2.4% were Asian. Eighty-three (82.8%) percent earned mostly A and B grades in school, while 29.5% were overweight or obese. Only 5.0% of the sample were part of the city of Philadelphia YRBS data collection while 7.6% reported rarely or never feeling safe in their neighborhood. Approximately one half of the sample (44.9%) reported at least 60-min of moderate physical activity on 5 or more days of the week. High sedentary behavior (≥4 h/day recreational screen time) was reported by 51.7% of the sample. (see Table 1 for a full listing of variables). These data are consistent with those reported by other data sources.

**Table 1:** Bivariate associations with Physical Activity (PA)

	Total sample *N* = 474,768 (*n* = 4020) CI = 405,872 - 543,663	High PA *N* = 213,443 (*n* = 1623) CI = 177,470 – 249,415	Low PA *N* = 261,324 (*n* = 2397) CI = 222,873 – 299,776	*χ* ^2^
Demographic variables				
Sex				
Female	50.5% (48.0% – 52.9%)	40.7% (38.0% – 43.6%)	58.4% (55.0% – 61.8%)	80.44***
Male	49.5% (47.1%–52.0%)	59.3% (56.4%– 62.0%)	41.6% (38.2% – 45.0%)	
Age				
< 16 years	40.5% (33.6% – 47.8%)	43.3% (35.5% – 51.4%)	38.2% (31.6% – 45.4%)	4.88*
≥ 16 years	59.5% (52.2% – 66.4%)	56.7% (48.6% – 64.5%)	61.8% (54.6% – 68.4%)	
Race				
Non–Hispanic white	70.9% (63.5% – 77.3%)	75.7% (69.1% – 81.2%)	67.0% (58.5% – 74.5%)	17.77***
Non–Hispanic black	15.2% (10.6% – 21.4%)	12.3% (8.2% – 17.9%)	17.6% (12.2% – 24.7%)	9.61**
Hispanic	8.4% (6.2% – 11.3%)	6.9% (4.9% – 9.6%)	9.6% (7.1% – 13.0%)	11.68***
Asian	2.4% (1.8% – 3.2%)	1.6% (1.0% –2.6%)	3.0% (2.2% – 4.1%)	7.25**
Other	3.1% (2.6% – 3.8%)	3.5% (2.6% – 4.7%)	2.8% (2.2% – 3.5%)	1.54
Academic achievement				
Earned mostly A’s & B’s	82.8% (79.3% – 85.7%)	85.2% (81.2%– 88.5%)	80.8% (77.0% – 84.1%)	6.34*
Overweight/obese	29.5% (27.2% – 32.0%)	25.3% (22.6% – 28.2%)	33.1% (30.3% – 36.0%)	23.62***
Environmental level variables				
Dataset (PA State vs. Philadelphia City)				
Philadelphia City	5.0% (3.9% – 6.3%)	3.6% (2.8% – 4.6%)	6.1% (4.7% – 7.8%)	34.26***
Perceived safety				
Rarely/never feel safe	7.6% (6.1% – 9.4%)	5.9% (4.6% – 7.7%)	8.9% (7.1% – 11.1%)	19.35***
Health related variables				
≥ 4 h screen time/day	51.7% (48.7% – 54.6%)	45.1% (40.8% – 49.3%)	57.1% (53.3% – 60.8%)	19.34***
≥ 1 day PE/week	58.6% (50.7% – 66.1%)	65.3% (56.9% – 72.7%)	53.2% (45.0% – 61.2%)	23.20***
≥ 1 sports teams	60.9% (57.9% – 63.8%)	79.4% (76.1% – 82.4%)	45.8% (42.6% – 49.0%)	258.78***
≥ 8 h sleep/night	25.6% (23.4% – 27.9%)	28.8% (25.7% – 32.0%)	23.1% (20.6% – 25.7%)	9.78**
Current alcohol use	30.6% (27.7% – 33.7%)	31.0% (27.4% – 34.9%)	30.3% (26.7% – 34.1%)	0.12
Current cigarette use	11.9% (9.7% – 14.6%)	11.8% (9.1% – 15.2%)	12.6% (9.4% – 15.3%)	0.02
≥ 2 servings fruit/day	28.9% (26.5% – 31.3%)	36.3% (33.0% – 39.7%)	22.8% (20.6% – 25.2%)	84.88***
≥ 3 servings veg/day	10.5% (8.8% – 12.4%)	15.2% (12.2% – 18.7%)	6.6% (5.2% – 8.4%)	31.43***

The association between each dichotomous independent variable and the dichotomous outcomes of physical activity (column 3 & 4) was examined using chi–square test of independence

*p ≤ .05 **p ≤ .01 ***p ≤ .001

### Variables associated with five or more days per week of physical activity

At the bivariate level, perceived safety was associated with physical activity such that adolescents who reported rarely or never feeling safe had a significantly higher proportion of low physical activity (8.9% reported low physical activity versus 5.9% who reported high physical activity; *χ* ^2^ = 19.35, *p* ≤ .001). High levels of physical activity was also significantly associated with being male, being younger than 16 years, self-identifying as non-Hispanic white, earning mostly A and B grades, not being overweight or obese, and not participating in the Philadelphia city data collection. In terms of other health-related variables, engaging in physical education class at least 1 day per week, participating in one or more sports teams, accruing eight or more hours of sleep, eating two or more servings or fruit and three or more servings of vegetables per day as well as not accruing four or more hours of recreational screen time per day were all significantly associated with high levels of physical activity (Table 1).

### Variables associated with recreational screen-based sedentary behavior

High levels (≥4 h/day) of recreational screen-based sedentary behavior was not significantly associated with perceptions of neighborhood safety (*χ* ^2^ = 0.51, *p* > .05). Being male, aged less than 16 years, self-identifying as non-Hispanic Black, being obese or overweight and residing in the city of Philadelphia were significantly associated with high levels (≥4 h/day) of recreational screen-based sedentary behavior (Table 2). High levels of physical activity (≥5 days of physical activity), playing on one or more sports teams and eating three or more servings of vegetables per day was associated with low levels of recreational screen-based sedentary behavior (Table 1).

**Table 2:** Bivariate associations with recreational screen based sedentary behavior

	≥4 Hours Recreational Screen time per Day *N* = 245,305; *n* = 2152 CI = 206,106 – 284,503	<4 Hours Recreational Screen–time per Day *N* = 229,463; *n* = 1868 CI = 194,206 – 264,719	*χ* ^2^
Demographic variables			
Sex			
Female	48.1% (44.8% – 51.3%)	53.0% (49.4% – 56.6%)	4.17*
Male	51.9% (48.7% – 55.2%)	47.0% (43.4 – 50.6%)	4.17*
Age			
< 16 years	42.9% (35.6% – 50.6%)	37.9% (30.8% – 45.7%)	
≥ 16 years	57.1% (49.4% – 64.4%)	62.1% (54.3% – 69.2%)	3.88*
Race			
Non–Hispanic white	67.8% (59.3% – 75.2%)	74.2% (67.6% – 79.8%)	12.67***
Non–Hispanic black	18.1% (12.5% – 25.5%)	12.1% (8.1% – 17.7%)	9.65**
Hispanic	9.2% (6.9% – 12.2%)	7.6% (5.2% – 10.8%)	3.57
Asian	1.7% (1.2% –2.4%)	3.1% (2.2% –4.3%)	10.28***
Other	3.2% (2.5% – 4.0%)	3.1% (2.3% – 4.1%)	0.02
Academic achievement			
Earned mostly A’s & B’s	78.4% (74.4% – 81.9%)	87.5% (83.6% – 90.5%)	21.28***
Overweight or obese	34.2% (31.1% – 37.5%)	24.6% (21.2% – 28.3%)	14.28***
Environmental level variables			
Philadelphia City	5.6% (4.3% – 7.1%)	4.4% (3.4% – 5.7%)	8.07**
Perceived safety			
Rarely/never feel safe	7.9% (6.3% – 9.9%)	7.2% (5.3% – 9.6%)	0.51
Health related variables			
≥ 5 days physical activity	39.5% (35.7% – 43.5%)	51.1% (47.2% – 55.0%)	19.34***
≥ 1 day PE/week	59.3% (51.3% – 66.9%)	57.8% (49.0% – 66.1%)	0.29
≥ 1 sports teams	55.0% (51.5% – 58.4%)	67.2% (63.1% – 71.0%)	27.09***
≥ 8 h sleep/night	25.0% (22.5% – 27.7%)	26.3% (23.3% – 29.6%)	0.55
Current alcohol use	29.5% (26.0% – 33.3%)	31.8% (28.0% – 35.8%)	0.97
Current cigarette use	12.4% (9.7% – 15.6%)	11.4% (9.0% – 14.4%)	0.47
≥ 2 servings fruit/day	27.2% (24.5% – 30.0%)	30.7% (27.5% – 34.0%)	3.38
≥ 3 servings veg/ day	8.6% (6.8% – 10.9%)	12.4% (10.5% – 14.7%)	12.30***

The association between each dichotomous independent variable and the dichotomous outcome of high vs. low recreational screen based sedentary behavior (column 2 & 3) was examined using chi–square test of independence

*p ≤ .05 **p ≤ .01 ***p ≤ .001

### Independent association between perceived safety and high physical activity

In a multivariable logistic regression model of high physical activity (≥ 5 days of activity per week) with perceived neighborhood safety as the independent variable of interest, adolescents who reported low levels of perceived safety had a 21% lower odds of being highly physical activity (OR = 0.79, CI = 0.56-0.89, *p* = .044) even after adjustment for other known determinants of physical activity (Table 3).

**Table 3:** Multivariable associations with physical activity on five or more days of the last week

	OR	95% Confidence Interval	*P*
Gender (1 = Male)	2.11	1.78 – 2.51	<0.001
Race (1 = Non-Hispanic White)	1.42	1.12 - 1.80	0.004
Academic achievement	0.94	0.73 – 1.21	0.640
Overweight or obese	0.87	0.72-1.04	0.129
City of Philadelphia only	1.11	0.82-1.48	0.499
Sports team participation	4.40	3.56-5.43	<0.001
≥ 8 or more hours of sleep	1.17	0.10-1.41	0.117
Adequate fruit consumption	1.62	1.36-1.92	<0.001
Adequate vegetable consumption	2.17	1.48-3.20	<0.001
High screen based sedentary behavior	0.69	0.54-0.88	<0.001
Low perceived safety	0.79	0.56-0.89	0.044

Other variables significantly associated with higher levels of physical activity included being male (OR = 2.11, CI = 1.78-2.51, *p* < 0.001), self-reporting as non-Hispanic white relative to all other racial and ethnic groups (OR = 1.42, CI = 1.12-1.80, *p* = 0.004), sports team participation (OR = 4.40, CI = 3.56-5.43, <0.001), adequate fruit consumption (OR = 1.62, CI = 1.36-1.92, p < 0.001), adequate vegetable consumption (OR = 2.17, CI = 1.48-3.20, p < 0.001). In contrast, high levels (≥ 4 h/day) of screen-based sedentary behavior was significantly associated with reduced odds of being active on five or more days per week (OR = 0.68, CI = 0.54-0.88, *p* < 0.001) (Table 3).

### Independent association between perceived safety and recreational screen-based sedentary behavior

In a multivariable model of high levels of recreational screen-based sedentary behavior, perceived safety did not emerge as a significant correlate, after adjustment for other significant correlates of recreational screen-based sedentary behavior. Instead, being male (OR = 1.25, CI = 1.01-1.53, *p* = 0.039) and being overweight or obese (OR = 1.39, CI = 1.10-1.75, *p* = 0.006) were associated with high levels of screen-based sedentary behavior. Additionally, high academic achievement (OR = 0.60,CI = 0.44-0.80, p < 0.001), sports team participation (OR = 0.73, CI = 0.52-0.86, *p* = 0.002), adequate vegetable consumption (OR = 0.67, CI = 0.52-0.86, p = 0.002) and high physical activity (OR = 0.67, CI = 0.54-0.85, *p* = 0.001) was independently associated with low recreational screen-based sedentary behavior (Table 4).

**Table 4:** Multivariable associations with high recreational screen based sedentary behavior (≥4 h/day)

	OR	95% Confidence interval	*P*
Gender (1 = Male)	1.25	1.01-1.53	0.039
Race (1 = Non-Hispanic White)	0.83	0.68-1.01	0.066
Academic achievement	0.60	0.44-0.80	<0.001
Overweight or obese	1.39	1.10-1.75	0.006
Physical Education class participation	1.18	0.94-1.49	0.152
Sports team participation	0.73	0.61-0.89	0.002
Adequate vegetable consumption	0.67	0.52-0.86	0.002
High physical activity	0.67	0.54-0.85	0.001
Low perceived safety	0.84	0.60-1.18	0.311

## Discussion

Increasing physical activity and reducing recreational screen-based sedentary behavior (i.e., television viewing and leisure computer use), are particularly important to improving cardiovascular health indices in youth and to forestall the incidence and progression of poor heart health in later years [38, 39]. Low neighborhood safety and perceptions of neighborhood safety are associated with lower levels of physical activity in adult populations [40]. In expanding on this literature to examine the association between recreational screen-based sedentary behavior with perceived safety in a population sample of adolescents, these data showed that while perceived safety was not associated with recreational screen-based sedentary behavior, it was significantly associated with physical activity. Specifically, youth who perceived their neighborhoods as unsafe had a 21% reduced odds of achieving five or more days of physical activity in the last week after adjustment for other known determinants of physical activity including sex, obesity status, and dietary intake. Other notable findings include data showing that participation in sports teams were strong correlates of sufficient physical activity and reduced recreational screen-based sedentary time. Together these population data suggest a role of perceived safety in physical activity promotion and underscore the importance of organized, formal physical activity opportunities for youth in achieving recommended levels of leisure-time physical activity and lower screen-based sedentary behavior.

In adults, high levels of neighborhood crime and low perceived safety have consistently been shown to relate to lower levels of physical activity [41]. Similarly, low levels of perceived neighborhood safety in parents is associated with lower levels of outdoor play and physical activity in their young children [42, 43], while high levels of perceived neighborhood safety correlated positively with child activity levels [44, 45]. Data have also shown children’s perceptions of neighborhood features (i.e., traffic density) to correspond with their parent’s perceptions [46]. Studies mostly conducted outside the US have reported adolescent perceptions of safety to be associated with physical activity levels [47, 48]. Increased fear of going outside to exercise [49], and lower feelings of community trust or social cohesion [50], are possible mechanisms that may help explain why low levels of perceived safety may be significantly associated with low levels of physical activity. Alternatively, higher levels of physical activity may lead to improved neighborhood perceptions. Evaluation of these, and other possible mechanisms for this association, is necessary in adolescents.

That perceived safety was significantly related to physical activity but not recreational screen-based sedentary behavior is consistent with data showing that physical activity and sedentary behavior in youth are not always related [51, 52]. In terms of other studies that have related perceived safety to sedentary behavior, data have shown that children whose parents perceived their neighborhood as unsafe had significantly higher levels of sedentary behavior than children whose parents perceived their neighborhood as safe [44, 53]. Our finding that adolescent perceived safety did not relate to recreational screen-based sedentary time diverges from this parental literature, and suggests that targeting parental perceived safety as opposed to youth perceived safety may be more important to reducing sedentary time in youth.

High levels of physical activity, sports team participation, and vegetable consumption emerged as being significantly related to reduced recreation screen-based sedentary behavior in this sample of youth. These data add to previous work showing increased physical activity [54], no participation on sports teams [55], and low vegetable consumption [56], to be associated with increased sedentary behavior. Together, this work aligns with a growing consideration of the clustering of health behaviors, and the recognition that negative health behaviors, such as poor diet and physical inactivity, tend to co-occur [57]. Addressing multiple risk factors in adolescents has demonstrated feasibility [58]; the current data provide candidate behaviors (i.e., physical activity, sports team participation and vegetable consumption) to be addressed in the context of reducing sedentary behavior.

The clinical and policy implications of these data are three-fold. First, these data reiterate the concept that sedentary behavior is not simply the other side of the physical activity coin, and instead is a separate construct that can and should be addressed independently [51]. Second, improving perceived neighborhood safety has been identified by these data as a potentially important intervention target to increase physical activity in youth. Changes to the physical and built environment may impact how spaces are used and by whom. For example, “greening” approaches (i.e., cleaning vacant lots, planting grass and trees, building a wooden fence around the perimeter) have been shown to hold multiple benefits for a community including increased feelings of perceived safety [59] and activity levels in urban adult residents [60]. Whether such an approach could increase levels of perceived safety and physical activity in adolescents has yet to be examined but such exploration holds relevance in the broader context of increasing community-level safety and physical activity. Third, these data highlight how critical organized physical activity and team sports are to youth activity levels. Concerning national trends include the drastic reductions in the required physical education class (i.e., 25% in 8th grade and 5% of 12th grade) [61] and declining quality of physical education instruction with only a third of adolescents being physically active in PE class for more than 20 min 3 to 5 days a week [62]. Efforts to improve perceived neighborhood safety and increase the requirement and quality of physical education class as well as promotion of sport team participation could incite far-reaching benefits for cardiovascular health in the short and longer term among youth [63].

While the current study is one of the largest population level examinations of the association between perceived safety in physical activity and recreational screen-based sedentary behavior in adolescents, these data should be interpreted with consideration to the fact that these data are based on self-report estimations and that several study constructs, including physical activity, was measured using single items. Importantly, observed neighborhood safety was not measured or considered in the multivariable models thus multilevel modeling based on neighborhood-level factors was not feasible. Physical activity and dietary habits were queried with the brief, standard YRBS questions rather than with longer construct-specific inventories. Finally, lack of sample homogeneity limits the generalizabity of these results. Despite these limitations, these data point to the importance of considering sedentary behavior and physical activity as separate entities and present sports team participation and perceived safety, respectively, as being possible intervention targets to achieve improvements in these cardiovascular risk behaviors in adolescents.

## Conclusions

Findings from this study of a large and diverse sample of adolescents add to a growing body of work demonstrating the importance of perceived safety with physical activity levels in youth. Perceived safety was associated with physical activity but not screen-based sedentary behavior, underscoring the notion that physical activity and sedentary behavior are distinct constructs. Sports team participation may be a viable target to reduce screen-based sedentary time among adolescents and should be assessed with future interventional research.
